# A modified delineation method of para‐aortic nodal clinical target volume in patients with locally advanced cervical cancer

**DOI:** 10.1002/cam4.4418

**Published:** 2021-11-16

**Authors:** Dunhuang Wang, Weiping Wang, Xiaoliang Liu, Kang Ren, Yongguang Liang, Qizhen Zhu, Fuquan Zhang, Ke Hu

**Affiliations:** ^1^ Department of Radiation Oncology, Peking Union Medical College Hospital Chinese Academy of Medical Sciences & Peking Union Medical College Beijing China

**Keywords:** cervical cancer, para‐aortic lymph node, para‐aortic nodal CTV, PET/CT

## Abstract

**Purpose:**

To validate the nodal center coverage (NCC) of the three mainstream delineation methods of para‐aortic nodal clinical target volume (CTV) and propose a modified delineation method of para‐aortic nodal CTV in prophylactic extended‐field irradiation (EFI) of cervical cancer.

**Methods:**

A total of 106 patients with para‐aortic lymph nodes (PALNs) identified on PET/CT were included at Peking Union Medical College Hospital between 2011 and 2020. PALNs were classified as left lateral para‐aortic (LLPA), aorto‐caval (AC), and right para‐caval (RPC). Distances from the nodal center to the aorta and inferior vena cava (IVC) were measured. The NCC of the three mainstream delineation methods of para‐aortic nodal CTV (CTV‐K, CTV‐S, and CTV‐D) and a modified CTV (CTV‐M) was calculated. Radiotherapy plans were created based on 4 CTVs for 10 selected patients who received prophylactic EFI. The chi‐squared test and the Student's *t*‐test were performed.

**Results:**

We identified 344 PALNs (216 LLPA, 101 AC, and 27 RPC) in 106 patients. Mean distance from the nodal center to the aorta was 9.6 mm in the LLPA and 7 mm in the AC and from the nodal center to the IVC was 5.6 mm in the AC and 5.6 mm in the RPC. CTV‐D improved the NCC of 98% compared with 92% for CTV‐K (*p *= 0.002) and 95% for CTV‐S (*p *= 0.046). CTV‐M provided the same satisfactory NCC as CTV‐D (97% vs. 98%, *p* = 0.485). The V_50Gy_ to the duodenum, the D_mean_ to the bilateral kidneys, and the V_45Gy_ to the small bowel were significantly lower on the CTV‐M‐based plan than on the CTV‐D‐based plan (*p *= 0.001, 0.011, and 0.001, respectively).

**Conclusion:**

CTV‐D provided more satisfactory NCC than CTV‐K and CTV‐S. CTV‐M provided the same satisfactory NCC as CTV‐D and reduced the dose to the critical structures.

## Graphical abstract

Our study is currently the largest data set in previous studies of anatomic distribution of fluorodeoxyglucose‐avid para‐aortic lymph nodes in cervical cancer. We validate nodal center coverage of the three mainstream delineation methods of para‐aortic nodal clinical target volume on our population and propose a modified delineation method to protect the critical structures.

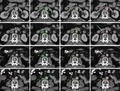

## 1 INTRODUCTION

Cervical cancer is a common gynecological malignancy worldwide, especially in transitioning countries. Globally, cervical cancer ranks fourth in terms of incidence (604000 new cases) and fourth in mortality overall (342000 deaths) in women in 2020.^1^ Pelvic and para‐aortic lymphatic regions are common sites of metastasis in patients with cervical cancer. Nomden et al. have reported that 52% of patients with cervical cancer had nodal involvement at diagnosis; of these, almost all had pelvic nodal involvement and 14% had para‐aortic nodal involvement.^2^ Similarly, a review of 22 studies found that 18% of patients with cervical cancer stages IB–IVA had para‐aortic nodal involvement.^3^ Additionally, Vandeperre et al. have reported that para‐aortic lymph node (PALN) metastases were present in 8% of cervical cancer patients with negative imaging at surgical staging.^4^ On the other hand, PALNs are common sites of recurrence after curative radiotherapy for cervical cancer.^2^, ^5^ Lymph node metastases negatively affected survival outcomes in patients with cervical cancer, particularly in patients with PALN metastases with a large survival decrement.^6^ According to the 2018 International Federation of Gynecology and Obstetrics (FIGO) staging system, patients with positive pelvic and para‐aortic lymph nodes are classified as stages IIIC1 and IIIC2, respectively.^7^

At present, extended‐field irradiation (EFI) is recommended as the standard treatment for patients with common iliac or para‐aortic nodal involvement. Additionally, recent studies suggested that prophylactic EFI should be performed for patients with high‐risk cervical cancer (such as pelvic wall invasion and pelvic nodal involvement) without evidence of para‐aortic nodal involvement, which could reduce the risk of para‐aortic nodal failure and provide potential benefits on survival outcomes.^8^, ^9^ On the contrast, some studies indicated that prophylactic EFI did not reduce the risk of para‐aortic nodal recurrence and confer survival benefits in patients with positive pelvic lymph node cervical cancer.^10^, ^11^ Anyway, prophylactic EFI is considered to be safe in modern radiotherapy techniques without a significant increase in severe toxicities in patients with cervical cancer.^11^, ^12^, ^13^

Currently, there are three mainstream delineation methods of the para‐aortic nodal clinical target volume (CTV) for intensity‐modulated radiation therapy in patients with cervical cancer and remain controversial. Keenan et al. mapped the PALNs for cervical cancer and proposed a contouring atlas for the para‐aortic nodal CTV expansion from the aorta of 10 mm anteriorly/posteriorly/medially and 15 mm laterally, and from the inferior vena cava (IVC) of 8 mm anteriorly/medially and 6 mm posteriorly/laterally to allow 97% nodal center coverage (NCC).^14^ Small et al. updated the guidelines of pelvic and para‐aortic nodal CTV delineations in endometrial and cervical cancer treated with postoperative radiotherapy.^15^ This para‐aortic nodal CTV should cover 10–20 mm left lateral to the aorta and extend to the medial border of the left psoas muscle, and 3–5 mm right lateral to the IVC and immediately anterior to the aorta and IVC. D’Cunha et al. proposed a modified para‐aortic nodal CTV, to optimize NCC, that is a blend of CTV delineations by Takiar et al., and Keenan et al.^14^, ^16^, ^17^ This CTV included 7 mm anterior to the aorta, left lateral para‐aortic posterior, and retrocaval regions between the level of the left renal vein and the level of the aortic bifurcation.

Our study was to validate para‐aortic NCC of the delineation methods by Keenan et al., Small et al., and D’Cunha et al., on our population and propose a modified delineation method of para‐aortic nodal CTV.

## 2 MATERIALS AND METHODS

### 2.1 Patients

We reviewed 181 consecutive patients with cervical cancer with positive PALNs identified on fluorodeoxyglucose (FDG) positron emission tomography/computed tomography (PET/CT) at Peking Union Medical College Hospital (PUMCH) between 2011 and 2020. Of these, 36 patients with stage IVB cervical cancer and 39 patients with para‐aortic nodal failure after initial treatment were excluded.

### 2.2 PET/CT examination

The patients prepared by fasting for at least 4 hours and controlling a blood glucose level of less than 8 mmol/L before an intravenous administration of ^18^F‐FDG (0.1–0.2 mCi/kg). The ^18^F‐FDG PET/CT was performed in a three‐dimensional mode with a scanning range from the skull base to the symphysis pubis using a Siemens Biograph 64 PET/CT scanner 60 min after injection. A low‐dose CT scan was acquired for attenuation and scatter correction and colocalization of radiologically equivalent interpretation.

### 2.3 CT simulation

The patients were prepared by emptying the rectum, filling the bladder, and using oral meglumine diatrizoate to visualize the bowel before CT simulation. The patients were in the head‐first supine position with thermoplastic material immobilization during the CT simulation. The CT simulation was performed with a scanning range from the diaphragm to the perineum and a slice thickness of 5 mm using a 16‐slice Philips Brilliance Big Bore CT simulator.

### 2.4 Lymph node distribution

Para‐aortic lymph nodes were classified as pathological if they presented higher FDG uptake than the background activity. The PET images were registered with a CT simulation scan, on which the PALNs were contoured for all patients. All positive PALNs were classified as left lateral para‐aortic (LLPA), aorto‐caval (AC), or right para‐caval (RPC) region, and upper (T12 to L1/L2 interspace), middle (L2 to L3/L4 interspace), or lower (L4 to aortic bifurcation) region based on the positions of the center of the lymph nodes relative to the vessels and the vertebral bodies. Additionally, the positive PALNs were also divided into A (coeliac trunk to left renal vein), B (left renal vein to inferior mesenteric artery), or C (inferior mesenteric artery to aortic bifurcation) region. The level of the renal vein, the inferior mesenteric artery, and the aortic bifurcation correspond with the vertebral body structures for all patients were recorded.

**FIGURE 1:**
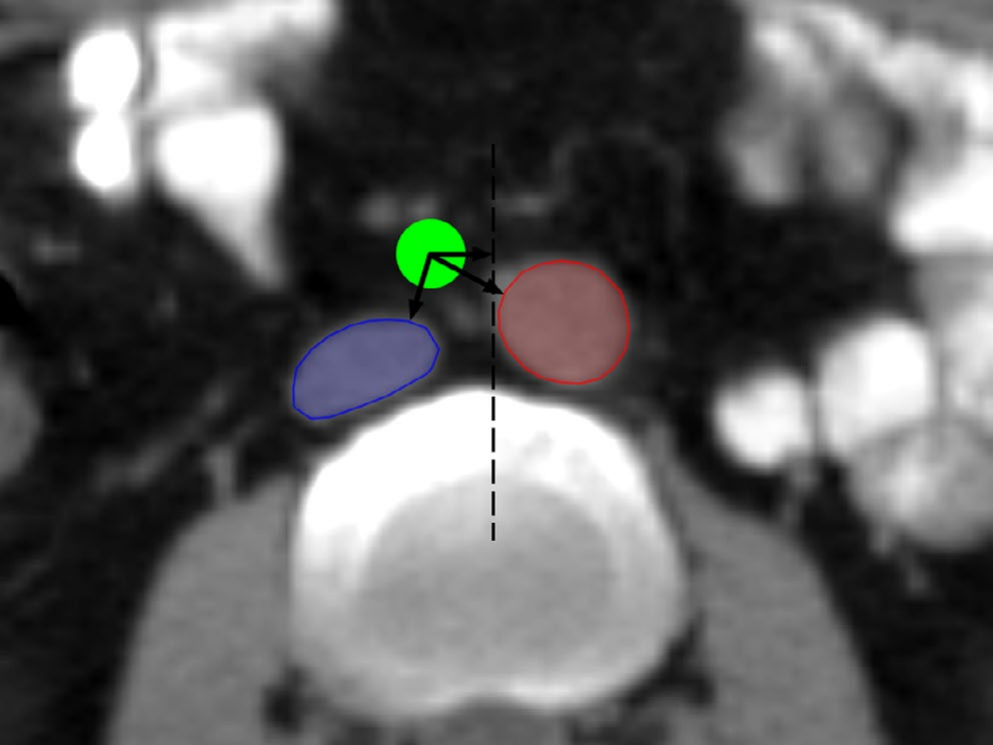
Distances to the center of the lymph node. The distances from the center of the lymph node (green) to the aorta (red) and IVC (blue) and to the midline (dotted line) of the vertebral body were measured. IVC, inferior vena cava

The distance from the center of the lymph node to the closest edge of the aorta and IVC was measured (Figure 1). The nodal center was defined as the geometric center of the largest plane of the lymph node on the axial CT image.

### 2.5 Contouring of para‐aortic nodal CTV

**FIGURE 2:**
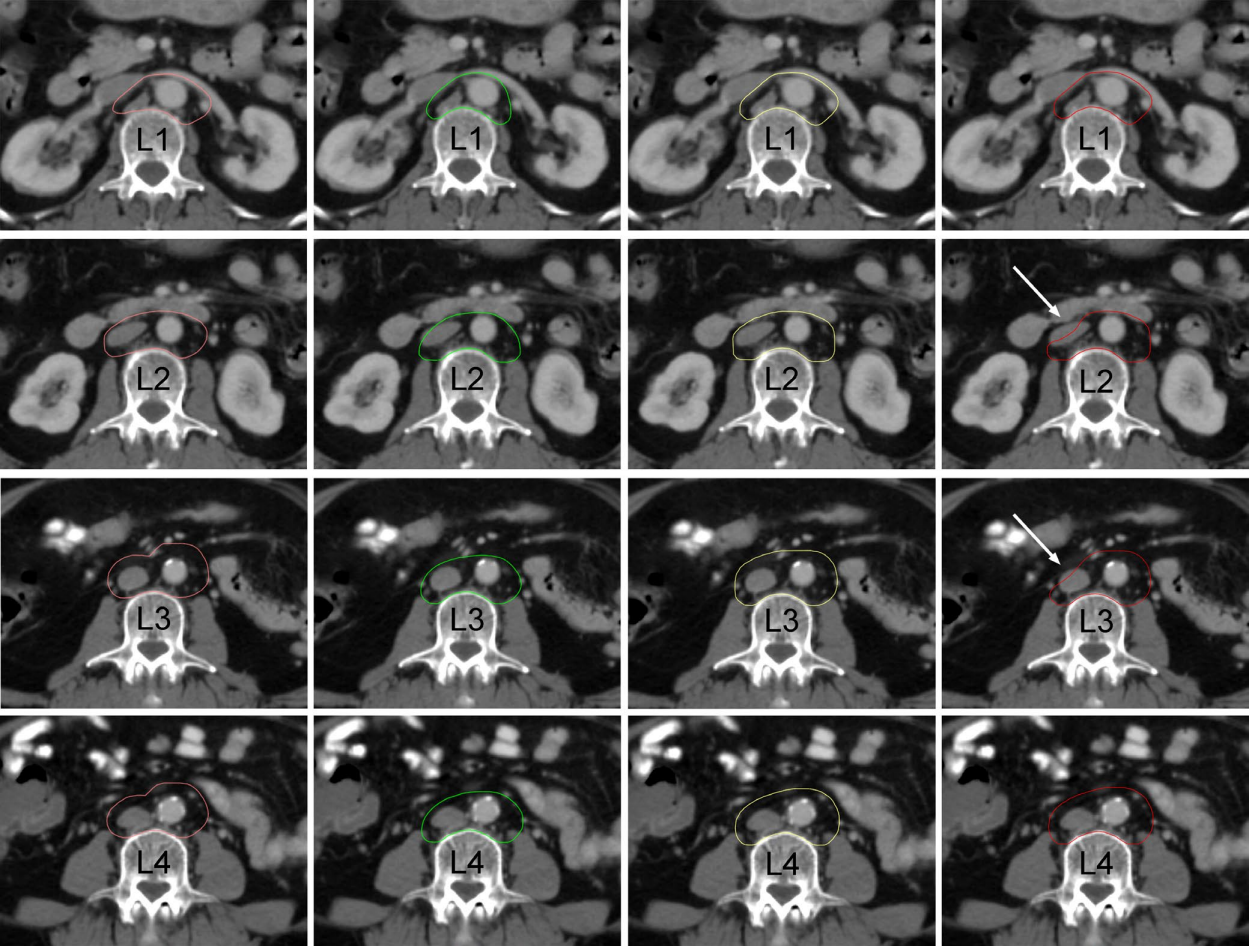
A modified delineation method of the para‐aortic nodal CTV, CTV‐M (red), with comparison to CTV‐K (pink), CTV‐S (green), and CTV‐D (yellow). Notably, the anterolateral region of RPC above the inferior mesenteric artery is omitted (white arrow) from CTV‐M to protect the duodenum. CTV, clinical target volume; RPC, right para‐caval

The contouring of para‐aortic nodal CTV was drawn based on the anatomic distribution of PALNs relative to the vessels in cervical cancer patients with para‐aortic nodal involvement. We contoured the para‐aortic nodal CTVs in three delineation methods based on the guidelines by Keenan et al. (CTV‐K), Small et al. (CTV‐S), and D’Cunha et al. (CTV‐D) for all patients with ignoring positive lymph nodes for prophylactic EFI.^14^, ^15^, ^17^ The superior border was at the level of the left renal vein, and the inferior border was at the level of the aortic bifurcation. Keenan et al. proposed that expansion margins from the aorta of 10 mm circumferentially and 15 mm laterally, and from the IVC of 8 mm anteromedially and 6 mm posterolaterally provided 97% coverage of the PALNs.^14^ Small et al. recommended expansion margins from the aorta of 10–20 mm laterally to the medial border of the left psoas muscle, and from the IVC of 3–5 mm circumferentially.^15^ D’Cunha et al. updated the atlas of the para‐aortic nodal CTV, which included 7 mm anterior to the aorta, LLPA posterior, and retrocaval coverage to provide optimal coverage of the PALNs.^17^ We also generated a modified CTV (CTV‐M), which omitted the anterolateral region of RPC above the inferior mesenteric artery to protect the duodenum (Figure 2). These four CTVs are described in Table 1.

**TABLE 1:** Guidelines for delineation of CTV‐K, CTV‐S, CTV‐D, and CTV‐M

	CTV‐K	CTV‐S	CTV‐D	CTV‐M
Superior border	The left renal vein	The left renal vein	The left renal vein	The left renal vein
Inferior border	The aortic bifurcation	The aortic bifurcation	The aortic bifurcation	The aortic bifurcation
Delineation	Expand the aorta by a margin of 10 mm anteriorly, posteriorly, and medially, and 15 mm laterally. Expand the IVC by 8 mm anteriorly and medially, and 6 mm posteriorly and laterally. Crop the CTV from normal boundaries such as the vertebral body, muscle, and bowel and expand the posterior border to the anterior vertebral body. Crop the CTV to exclude the RPC region above the L1–L2 interspace.	A noncontrast CT should be used for CTV delineation. This CTV should extend laterally from the aorta to the medial border of the left psoas muscle, which typically places the left lateral border 1–2 cm lateral to the aorta. On the right, the CTV margin is typically within 3–5 mm around the IVC. There is minimal evidence of nodal involvement to the right of the IVC and immediately anterior to the aorta and IVC where margins should be tighter. The space between the aorta and IVC should be straight.	A modified CTV, CTV‐D, that is a blend of CTV‐K and CTV‐T (Takair et al.^16^). This CTV includes a 7 mm anterior aortic expansion in the absence of bowel, LLPA posterior, and retrocaval coverage from the renal vein to the aortic bifurcation.	A modified CTV, CTV‐M, that is a modification of CTV‐D. The anterolateral region of RPC above the inferior mesenteric artery is omitted from the para‐aortic nodal CTV.

Abbreviations: CT, computed tomography; CTV, clinical target volume; IVC, inferior vena cava; LLPA, left lateral para‐aortic; RPC, right para‐caval.

### 2.6 Calculation of NCC and dosimetric comparison of organs at risk (OARs)

The NCC of the four CTVs was calculated. The calculation formula of the NCC is as follows:

$${\mathrm{NCC}}_{\#}=\frac{n_{\#}}{N_{\#}}*100\%$$

where *n* is the number of the lymph nodes in the CTV, *N* is the number of the lymph nodes in the lymph node region, and # represents the lymph node region (LLPA, AC, RPC, or whole PALN region). Additionally, radiotherapy plans were created based on four CTVs for 10 selected patients who received prophylactic EFI. A 7–10 mm margin was added to CTV to create the planning target volume (PTV) for setup errors and other uncertainties. External beam radiotherapy delivered a dose prescription of 50.4 Gy in 28 fractions (1.8 Gy /fraction) to at least 95% of the PTV, using volumetric‐modulated arc therapy (VMAT) on a TrueBeam system (version 2.7; Varian Medical Systems). The dose constraints of the upper abdominal OARs were as follows: duodenum, V_55Gy_ ≤ 1 cm^3^; bilateral kidneys, D_mean_ ≤ 18 Gy; small bowel, V_45Gy_ ≤ 275 cm^3^; and spinal cord, D_0.1cc_ ≤ 45 Gy.^18^, ^19^, ^20^

### 2.7 Statistics

The SPSS version 26 (IBM Corp.) was applied for all statistical analyses. The distance from the nodal center to the aorta and the IVC was summarized using the average and the standard deviation (SD). The chi‐squared test or the Fisher's exact test was used to compare the NCC between the four CTVs, and the Student's *t*‐test was performed to compare the dose to the OARs between the four plans. A two‐sided *p* value less than 0.05 was considered to be statistically significant.

## 3 RESULTS

The characteristics of the included patients are shown in Table 2. Median age of the 106 included patients was 51 years (range, 25–82 years). Eighty‐four patients had squamous cell carcinoma, 14 had adenocarcinoma, 2 had adenosquamous carcinoma, 1 had carcinosarcoma, 1 had high‐grade serous carcinoma, and 4 had undifferentiated carcinoma. One hundred and one (95%) patients were classified as 2018 FIGO stage IIIC2 and 5 (5%) patients were classified as 2018 FIGO stage IVA. Additionally, 7 (7%), 5 (5%), 51 (48%), 1 (1%), 37 (35%), and 5 (5%) patients were classified as 2009 FIGO stages IB, IIA, IIB, IIIA, IIIB, and IVA, respectively. All patients were treated with definitive radiotherapy or chemoradiotherapy.

**TABLE 2:** Characteristics of the included patients

Characteristic	No. of patients	Percent of patients
Median age, year	51 (range, 25–82)	
Median no. of PALNs	3 (range, 1–11)	
2009 FIGO stages		
IB	7	7%
IIA	5	5%
IIB	51	48%
IIIA	1	1%
IIIB	37	35%
IVA	5	5%
2018 FIGO stages		
IIIC2	101	95%
IVA	5	5%
Histology		
Squamous cell carcinoma	84	79%
Adenocarcinoma	14	13%
Adenosquamous	2	2%
Carcinosarcoma	1	1%
Serous carcinoma	1	1%
Undifferentiated carcinoma	4	4%
Primary tumor size		
≤4 cm	35	33%
>4 cm	71	67%
PLNs		
No	5	5%
Yes	101	95%

Abbreviations: FIGO, International Federation of Gynecology and Obstetrics; No., number; PALNs, para‐aortic lymph nodes; PLNs, pelvic lymph nodes.

**FIGURE 3:**
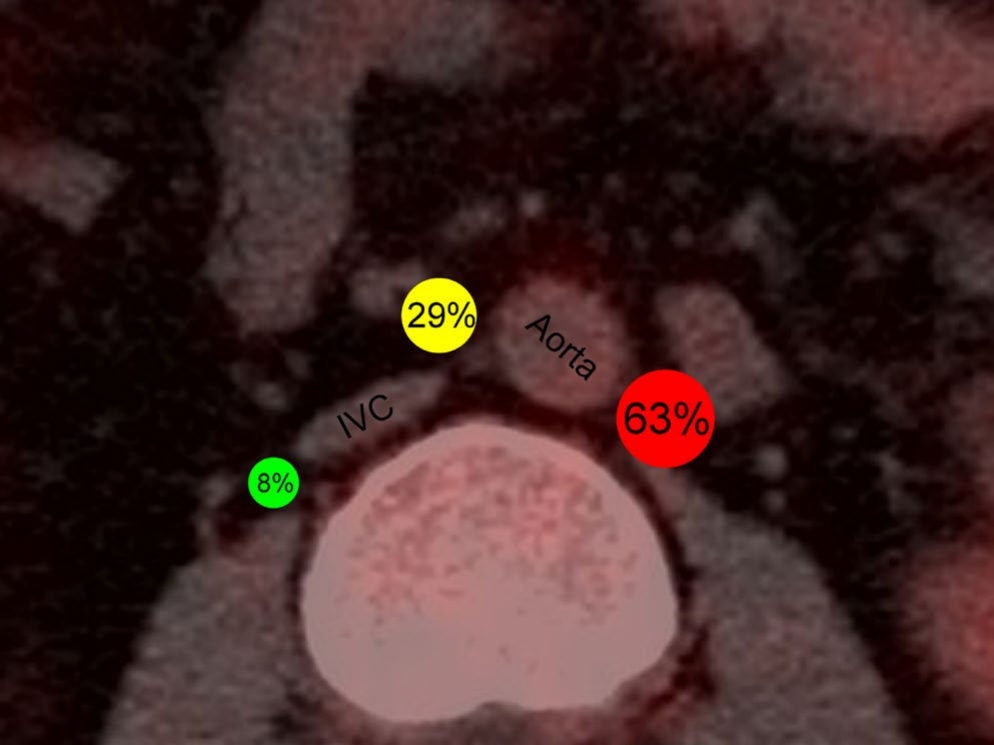
Anatomic distribution of PALNs in the axial plane. The anatomic distribution of PALNs in the LLPA (red), AC (yellow), and RPC (green) regions was calculated. AC, aorto‐caval; LLPA, left lateral para‐aortic; PALN, para‐aortic lymph node; RPC, right para‐caval

A total of 344 FDG‐avid PALNs on PET/CT were identified in 106 included patients with locally advanced cervical cancer. Mean (SD) minor axis of PALNs was 9 (2.8) mm (range, 5–23 mm). Of the 344 PALNs, 63% (*n* = 216) were in the LLPA region, 29% (*n* = 101) were in the AC region, and 8% (*n* = 27) were in the RPC region (Figure 3). Additionally, 6% (*n* = 19) of PALNs were in the upper region, 74% (*n* = 254) were in the middle region, and 21% (*n* = 71) were in the lower region. Besides, 64% (*n* = 220) were to the left of the midline of the vertebral body, 1% (*n* = 3) were at the midline of the vertebral body, and 35% (*n* = 121) were to the right of the midline of the vertebral body. The anatomic distribution of PALNs is shown in Table 3 with comparison to Takiar et al., Keenan et al., and D’Cunha et al.^14^, ^16^, ^17^

**TABLE 3:** Anatomic distribution of PALNs with comparison to previous studies

	Our study	Takiar et al.^16^	Keenan et al.^14^	D’Cunha et al.^17^
No. of PALNs	344	72	68	176
LLPA	216 (63%)	37 (51%)	34 (50%)	94 (54%)
AC	101 (29%)	32 (44%)	29 (43%)	71 (40%)
RPC	27 (8%)	3 (4%)	5 (7%)	11 (6%)
Upper	19 (6%)	5 (7%)	2 (3%)	2 (1%)
Middle	254 (74%)	53 (74%)	46 (68%)	131 (74%)
Lower	71 (21%)	14 (19%)	20 (29%)	43 (24%)
A	6 (2%)	—	—	1 (1%)
B	159 (46%)	—	—	82 (47%)
C	179 (52%)	—	—	93 (53%)

Abbreviations: A, coeliac trunk to left renal vein; AC, aorto‐caval; B, left renal vein to inferior mesenteric artery; C, inferior mesenteric artery to aortic bifurcation; LLPA, left lateral para‐aortic; PALN, para‐aortic lymph node; RPC, right para‐caval.

**FIGURE 4:**
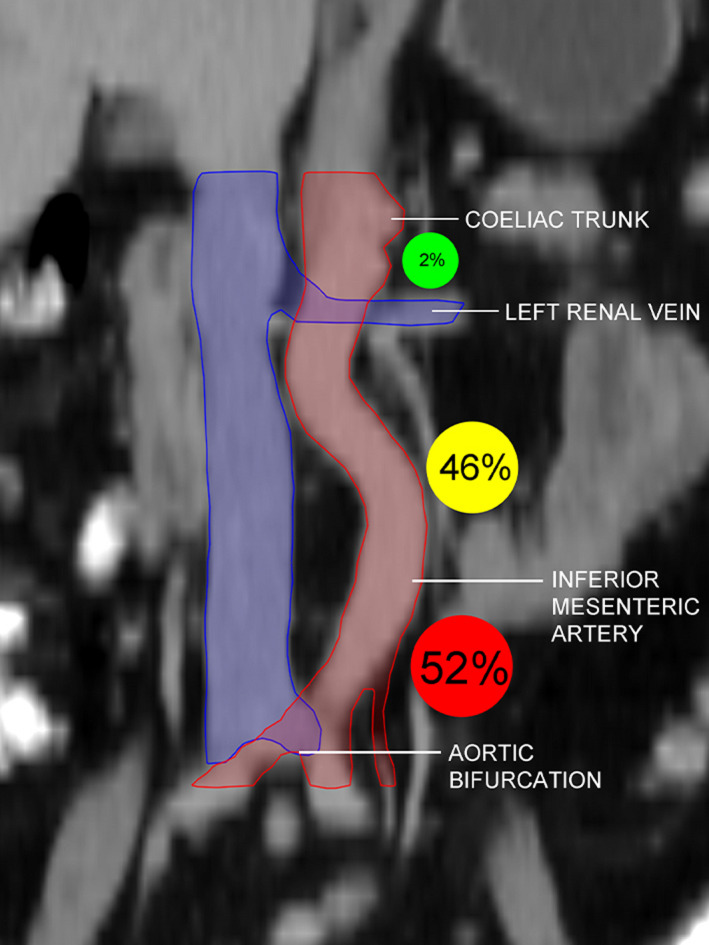
Anatomic distribution of PALNs in the coronal plane. The anatomic distribution of PALNs in the A (coeliac trunk to left renal vein, green), B (left renal vein to inferior mesenteric artery, yellow), and C (inferior mesenteric artery to aortic bifurcation, red) regions was calculated. Abbreviation: PALN, para‐aortic lymph node

The anatomic distribution of PALNs in each axial and coronal region is shown in Table 4. Of the 344 PALNs, 179 (100 LLPA, 60 AC, and 19 RPC) were between the level of the inferior mesenteric artery and the level of the aortic bifurcation, 159 (112 LLPA, 40 AC, and 7 RPC) were between the level of the left renal vein and the level of the inferior mesenteric artery, and 6 (4 LLPA, 1 AC, and 1 RPC) were between the level of the coeliac trunk and the level of the left renal vein. Notably, 98% of PALNs were below the level of the renal vein and less than 2% were above this level (Figure 4). Additionally, only 1% (3/344) of PALNs classified as RPC were in the anterolateral region and all of these were below the level of the inferior mesenteric artery.

**TABLE 4:** Anatomic distribution of PALNs in each axial and coronal region

	LLPA	AC	RPC
Upper	16 (5%)	3 (1%)	0 (0)
Middle	166 (48%)	73 (21%)	15 (4%)
Lower	34 (10%)	25 (7%)	12 (3%)
A	4 (1%)	1 (0)	1 (0)
B	112 (33%)	40 (12%)	7 (2%)
C	100 (29%)	60 (17%)	19 (6%)

Abbreviations: A, coeliac trunk to left renal vein; AC, aorto‐caval; B, left renal vein to inferior mesenteric artery; C, inferior mesenteric artery to aortic bifurcation; LLPA, left lateral para‐aortic; PALN, para‐aortic lymph node; RPC, right para‐caval.

The anatomic distribution of PALNs concerning the stage or clinical factors is shown in Table 5. Nine (60%), 108 (61%), 89 (66%), and 10 (59%) PALNs in the LLPA region were corresponding to the 2009 FIGO stages I, II, III, and IV; 6 (40%), 56 (31%), 32 (24%), and 7 (41%) PALNs in the AC region were corresponding to the 2009 FIGO stages I, II, III, and IV; and 0, 14 (8%), 13 (10%), and 0 PALNs in the RPC region were corresponding to the 2009 FIGO stages I, II, III, and IV, respectively. Fifteen (100%), 173 (97%), 133 (99%), and 17 (100%) PALNs below the level of the renal vein were corresponding to the 2009 FIGO stages I, II, III, and IV, respectively.

**TABLE 5:** Anatomic distribution of PALNs concerning the clinical factors

	2009 FIGO stage				Histology			Primary tumor size	
	Stage I	Stage II	Stage III	Stage IV	SCC	AD	Others	≤4 cm	>4 cm
No. of patients	7 (7%)	56 (53%)	38 (35%)	5 (5%)	84 (79%)	14 (13%)	8 (8%)	35 (33%)	71 (67%)
No. of PALNs	15 (4%)	178 (52%)	134 (39%)	17 (5%)	281 (82%)	41 (12%)	22 (6%)	108 (31%)	236 (69%)
Median no. of PALNs	2 (range, 1–3)	3 (range, 1–10)	3 (range, 1–11)	3 (range, 1–7)	3 (range, 1–11)	2 (range, 1–9)	3 (range, 1–4)	2 (range, 1–11)	3 (range, 1–10)
LLPA	9 (60%)	108 (61%)	89 (66%)	10 (59%)	173 (62%)	26 (63%)	17 (77%)	68 (63%)	148 (63%)
AC	6 (40%)	56 (31%)	32 (24%)	7 (41%)	83 (30%)	13 (32%)	5 (23%)	27 (25%)	74 (31%)
RPC	0	14 (8%)	13 (10%)	0	25 (9%)	2 (5%)	0	13 (12%)	14 (6%)
Upper	0	10 (6%)	8 (6%)	1 (6%)	15 (5%)	3 (7%)	1 (5%)	7 (6%)	12 (5%)
Middle	14 (93%)	131 (74%)	98 (73%)	11 (65%)	207 (74%)	31 (76%)	16 (73%)	72 (67%)	182 (77%)
Lower	1 (7%)	37 (21%)	28 (21%)	5 (29%)	59 (21%)	7 (17%)	5 (23%)	29 (27%)	42 (18%)
A	0	5 (3%)	1 (1%)	0	6 (2%)	0	0	1 (1%)	5 (2%)
B	8 (53%)	84 (47%)	62 (46%)	5 (29%)	131 (47%)	20 (49%)	8 (36%)	53 (49%)	106 (45%)
C	7 (47%)	89 (50%)	71 (53%)	12 (71%)	144 (51%)	21 (51%)	14 (64%)	54 (50%)	125 (53%)

Abbreviations: A, coeliac trunk to left renal vein; AC, aorto‐caval; AD, adenocarcinoma; B, left renal vein to inferior mesenteric artery; C, inferior mesenteric artery to aortic bifurcation; FIGO, International Federation of Gynecology and Obstetrics; LLPA, left lateral para‐aortic; PALN, para‐aortic lymph node; RPC, right para‐caval; SCC, squamous cell carcinoma.

The level of the left renal vein, the inferior mesenteric artery, and the aortic bifurcation at the vertebral body varied among patients with the left renal vein at the T12/L1 interspace for 5 (5%) patients, the L1 for 34 (32%) patients, the L1/L2 interspace for 48 (45%) patients, or the L2 for 19 (18%) patients, and with the inferior mesenteric artery at the L2 for 3 (3%) patients, the L2/L3 interspace for 30 (28%) patients, the L3 for 59 (56%) patients, the L3/L4 interspace for 13 (12%), or the L4 for 1 (1%) patient, and with the aortic bifurcation at the L3/L4 interspace for 20 (19%) patients, the L4 for 56 (53%) patients, the L4/L5 interspace for 27 (25%) patients, or the L5 for 3 (3%) patients.

The mean (SD) distance from the nodal center to the aorta was 9.6 (3.5) mm for the LLPA region and 7.0 (2.5) mm for the AC region, and from the nodal center to the IVC was 5.6 (2.1) mm for the AC region and 5.6 (2.0) mm for the RPC region, and from the nodal center to the midline of the vertebral body was 19.7 (5.1) mm for left‐sided nodes and 11.3 (8.2) mm for right‐sided nodes.

**FIGURE 5:**
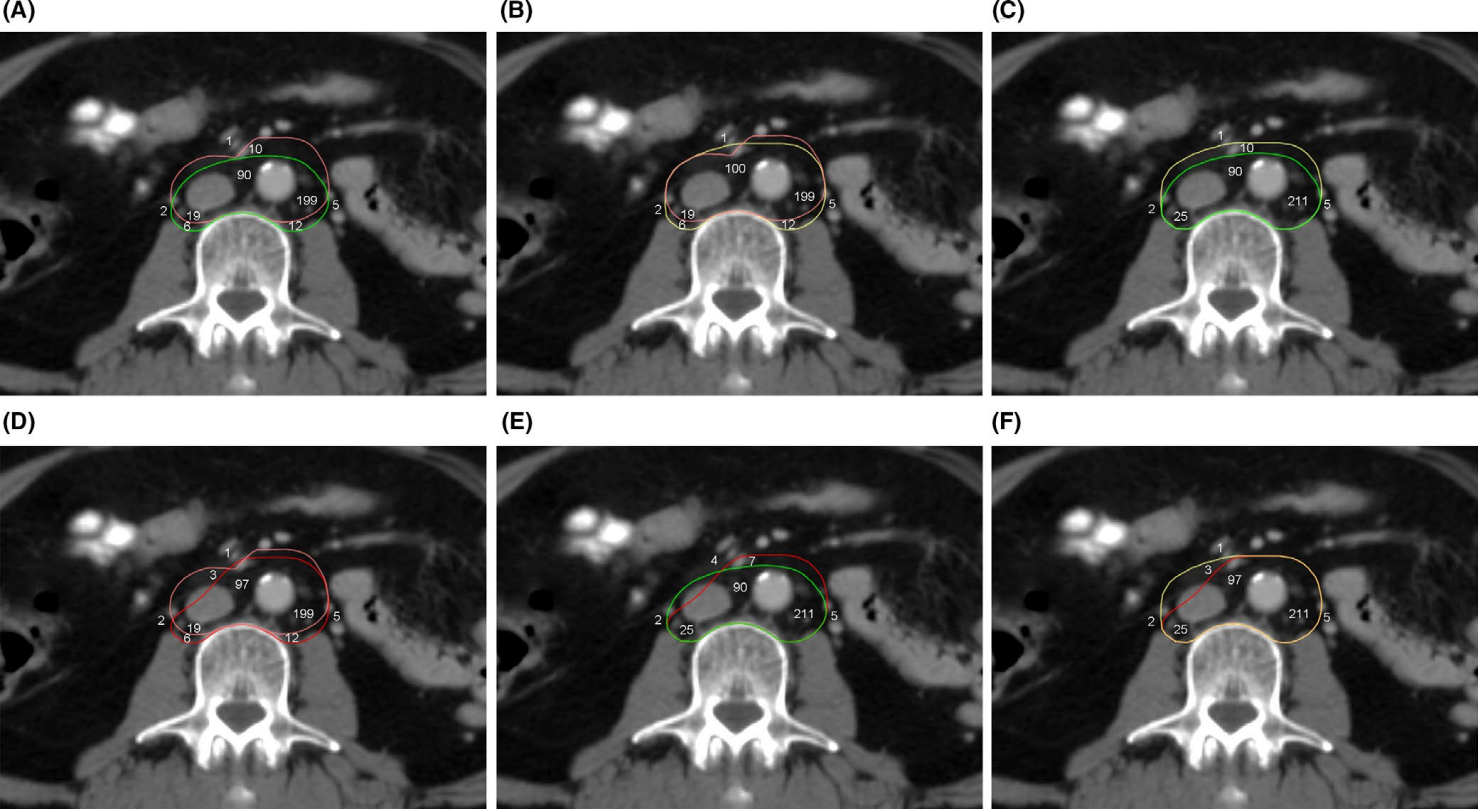
Comparison of the NCC between CTV‐K (pink), CTV‐S (green), CTV‐D (yellow), and CTV‐M (red). (A) CTV‐K versus CTV‐S, 92% versus 95%, *p* = 0.213; (B) CTV‐K versus CTV‐D, 92% versus 98%, *p *= 0.002; C: CTV‐S versus CTV‐D, 95% versus 98%, *p* = 0.046; (D) CTV‐K versus CTV‐M, 92% versus 97%, *p* = 0.011; (E) CTV‐S versus CTV‐M, 95% versus 97%, *p *= 0.184; and (F) CTV‐D versus CTV‐M, 98% versus 97%, *p *= 0.485. The numerical value represents the number of the lymph nodes, and the location of the numerical value represents the location of the lymph nodes. The numerical value inside the CTV indicates that the nodal center is covered by the CTV, and the numerical value outside the CTV indicates that the nodal center is not covered by the CTV. CTV, clinical target volume; NCC, nodal center coverage

CTV‐D improved the para‐aortic NCC of 98% (336/344) compared with 92% (318/344) for CTV‐K (*p *= 0.002) and 95% (326/344) for CTV‐S (*p *= 0.046). Para‐aortic NCC for CTV‐D and CTV‐K was 98% (211/216) and 92% (199/216) in the LLPA region (*p* = 0.009), 99% (100/101) and 99% (100/101) in the AC region (*p *= 1), and 93% (25/27) and 70% (19/27) in the RPC region, and for CTV‐D and CTV‐S was 98% (336/344) and 98% (336/344) in the LLPA region (*p *= 1), 99% (100/101) and 89% (90/101) in the AC region, and 93% (25/27) and 93% (25/27) in the RPC region (*p *= 1), respectively. CTV‐M provided the same satisfactory NCC as CTV‐D (97% vs. 98%, *p* = 0.485). There were no significant differences in the LLPA, AC, and RPC regions (Table 6 and Figure 5).

**TABLE 6:** Comparison of the NCC between CTV‐K, CTV‐S, CTV‐D, and CTV‐M

	Total NCC	LLPA NCC	AC NCC	RPC NCC
CTV‐K	92% (318/344)	92% (199/216)	99% (100/101)	70% (19/27)
CTV‐S	95% (326/344)	98% (211/216)	89% (90/101)	93% (25/27)
CTV‐D	98% (336/344)	98% (211/216)	99% (100/101)	93% (25/27)
CTV‐M	97% (333/344)	98% (211/216)	96% (97/101)	93% (25/27)
P (CTV‐K vs. CTV‐S)	0.213	**0.009**	**0.003**	**0.036**
P (CTV‐K vs. CTV‐D)	**0.002**	**0.009**	1	**0.036**
P (CTV‐S vs. CTV‐D)	**0.046**	1	**0.003**	1
P (CTV‐K vs. CTV‐M)	**0.011**	**0.009**	0.365	**0.036**
P (CTV‐S vs. CTV‐M)	0.184	1	0.06	1
P (CTV‐D vs. CTV‐M)	0.485	1	0.365	1

Abbreviations: AC, aorto‐caval; CTV, clinical target volume; LLPA, left lateral para‐aortic; NCC, nodal center coverage; RPC, right para‐caval. Notably, the superior border is at the level of the left renal vein because there are hardly any lymph nodes above this level.

The bold values means that a two‐sided *p* value less than 0.05 is considered to be statistically significant.

The dosimetric comparison of the OARs between the four radiotherapy plans is shown in Table 7. For the duodenum, the V_50Gy_ was significantly lower on the CTV‐M than on the CTV‐K (10.7 cm^3^ vs. 18.0 cm^3^, *p *= 0.003), CTV‐S (10.7 cm^3^ vs. 14.0 cm^3^, *p* = 0.004), or CTV‐D (10.7 cm^3^ vs. 18.6 cm^3^, *p *= 0.001). For the bilateral kidneys, the D_mean_ was significantly lower on the CTV‐M than on the CTV‐D (12.1 Gy vs. 12.6 Gy, *p *= 0.011), while there was no difference between CTV‐M and CTV‐K or CTV‐S. And no difference was found in the V_20Gy_ to the bilateral kidneys between the four radiotherapy plans. For the small bowel, the V_45Gy_ was significantly lower on the CTV‐M than on the CTV‐K (224.3 cm^3^ vs. 248.9 cm^3^, *p *= 0.001) or CTV‐D (224.3 cm^3^ vs. 232.4 cm^3^, *p *= 0.001), while there was no difference between CTV‐M and CTV‐S.

**TABLE 7:** Dosimetric comparison of the OARs between CTV‐K, CTV‐S, CTV‐D, and CTV‐M

		CTV‐K (mean ± SD)	CTV‐S (mean ± SD)	CTV‐D (mean ± SD)	CTV‐M (mean ± SD)	P	P	P	P	P	P
						CTV‐K vs. CTV‐S	CTV‐K vs. CTV‐D	CTV‐S vs. CTV‐D	CTV‐K vs. CTV‐M	CTV‐S vs. CTV‐M	CTV‐D vs. CTV‐M
Duodenum	V_50Gy_ (cm^3^)	18.0 ± 7.0	14.0 ± 4.8	18.6 ± 6.7	10.7 ± 4.5	**0.010**	0.257	**0.002**	**0.003**	**0.004**	**0**.**001**
Bilateral kidneys	D_mean_ (Gy)	12.6 ± 1.5	11.3 ± 2.1	12.6 ± 2.8	12.1 ± 2.6	**0.005**	0.965	0.160	0.428	0.355	**0.011**
Bilateral kidneys	V_20Gy_ (%)	12.9 ± 4.2	10.2 ± 4.5	13.3 ± 6.2	11.8 ± 5.3	**0.003**	0.787	0.118	0.422	0.283	0.100
Small bowel	V_45Gy_ (cm^3^)	248.9 ± 68.3	223.6 ± 67.4	232.4 ± 59.2	224.3 ± 60.2	**0.002**	**0.016**	0.050	**0.001**	0.869	**0.001**

Abbreviations: CTV, clinical target volume; OARs, organs at risk.

The bold values means that a two‐sided *p* value less than 0.05 is considered to be statistically significant.

## 4 DISCUSSION

Pelvic and para‐aortic lymph nodes are major sites of metastasis and recurrence for cervical cancer, so the assessment of lymph node status plays an important role in diagnosis and prognosis of patients with cervical cancer.^21^ Patients with positive PALNs have worse survival outcomes with comparison to those without para‐aortic nodal involvement. Cervical cancer patients with high‐risk factors, such as pelvic lymph node metastases and tumor extension to the pelvic wall, may benefit from prophylactic EFI without a significant increase in severe adverse effects.^8^, ^9^, ^22^, ^23^ At PUMCH, a multicenter, prospective, randomized, controlled phase III trial of the efficacy and safety of prophylactic EFI with comparison to pelvic irradiation in patients with high‐risk cervical cancer is ongoing (NCT03955367).

Generally for cervical cancer, the para‐aortic nodal CTV refers to the lymph node regions at risk adjacent to the aorta and IVC from the superior border of the level of the left renal vein to the aortic bifurcation. The lymphatic fatty tissue of para‐aortic region was divided into two parts (the renal vessels to the inferior mesenteric artery and the inferior mesenteric artery to the aortic bifurcation) by the inferior mesenteric artery. Moreover, the lymphatic fatty tissue of this region was further divided into eight parts (para‐aortic, pre‐aortic, retro‐aortic, intercavo‐aortic superficial, intercavo‐aortic deep, paracaval, precaval, and retrocaval) based on the positions of the lymph nodes relative to the aorta and IVC.^21^ The delineation of the para‐aortic nodal CTV in cervical cancer is based on the anatomic distribution of the PALNs relative to the aorta and IVC.

However, guidelines regarding the para‐aortic nodal CTV delineations in cervical cancer are limited. At present, there are three mainstream guidelines of the para‐aortic nodal CTV in cervical cancer by Keenan et al., Small et al., and D’Cunha et al.^14^, ^15^, ^17^ However, the jury is still out on which of these guidelines is better. Our study is currently the largest data set in previously published studies of anatomic distribution of FDG‐avid PALNs in cervical cancer. We found that CTV‐D (98%) provided satisfactory NCC compared with CTV‐K (92%, *p *= 0.002) and CTV‐S (95%, *p *= 0.046) on our population. Compared with CTV‐K, CTV‐D significantly improved LLPA and RPC NCC. Compared with CTV‐S, CTV‐D significantly improved AC NCC.

In addition, we proposed a modified delineation method of para‐aortic nodal CTV, CTV‐M, based on CTV‐D to protect the duodenum and right kidney with satisfactory NCC (97% vs. 98%, *p *= 0.485). There are several similarities. First, the superior border of CTV‐M is at the level of the renal vein because hardly any positive lymph nodes are discovered above this level. Takiar et al. and Keenan et al. reported that there were no PALNs identified above the level of the renal vein.^14^, ^16^ D’Cunha et al. indicated that only 1 PALN out of the 176 was found above this level.^17^ Similarly, less than 2% (6/344) of the PALNs were found above the level of the renal vein, and all of these were below the level of the coeliac trunk in our study. Second, CTV‐M with a 7 mm anterior to the aorta the same as CTV‐D confers satisfactory coverage of these anterior lymph nodes. Similarly, in the EMBRACE II study, guidelines for delineation of para‐aortic nodal CTV recommend a 7 mm anterior to the vessels.^24^ Finally, CTV‐M includes 1–2 cm lateral to the aorta extension to the medial border of the left psoas muscle and posterolateral to the IVC extension to the medial border of the right psoas muscle to provide optimal NCC in these regions.

Notably, the major difference between CTV‐M and CTV‐D is that the anterolateral region of RPC above the inferior mesenteric artery is omitted from the CTV‐M to protect the duodenum. Because we found that there were no PALNs in the anterolateral region of RPC above the inferior mesenteric artery. Similarly, Takiar et al. found that all right para‐caval lymph nodes were located at or below L3, and the concept of limited anterior margin for the right para‐caval nodes had already been described in the published article by Small et al.^15^, ^16^ Additionally, since the duodenum is anterolateral and close to the IVC, omission of the anterolateral region of RPC can reduce the irradiation dose to the duodenum. Yang et al. demonstrated that for cervical cancer patients treated with prophylactic EFI, omission of the right para‐caval region above L3 from the para‐aortic nodal CTV could significantly reduce the irradiation dose to the duodenum in high‐dose area and may further reduce the incidence of duodenal toxicity.^25^

The duodenum, kidneys, and small bowel are dose‐limited structures in radiotherapy of gynecological cancers and abdominal cancers. For the duodenum, previous studies have found that the V_50Gy_ ≥ 4 cm^3^ and V_55Gy_ ≥ 1 cm^3^ or ≥15 cm^3^ were associated with duodenal toxicity in radiotherapy of gynecological cancers and abdominal cancers.^18^, ^26^, ^27^ Noteworthy, there was a significant difference in the V_50Gy_ to the duodenum between the CTV‐D and CTV‐M (*p *= 0.001). Additionally, in our published study of 20 patients with locally advanced cervical cancer who received prophylactic EFI using a similar delineation method of para‐aortic nodal CTV‐M, no patients experienced PALN recurrence at a median follow‐up of 26.5 months, and 5 (25%) patients experienced grade 3 acute gastrointestinal toxicity and 1 (5%) patient experienced grade 3 chronic gastrointestinal toxicity.^25^ For the bilateral kidneys, published studies have found that the D_mean_ and V_20Gy_ were associated with renal toxicity in partial‐body radiotherapy of gynecological cancers and abdominal cancers.^28^, ^29^ Noteworthy, there was a significant difference in the D_mean_ to the bilateral kidneys between CTV‐D and CTV‐M (*p *= 0.011). For the small bowel, published studies have found that the V_45Gy_ was associated with small bowel toxicity in radiotherapy of gynecological cancers.^20^, ^30^ Noteworthy, there was a significant difference in the V_45Gy_ to the small bowel between CTV‐D and CTV‐M (*p* = 0.001). Future studies are required to validate the correlation between the dose data and the risk of the critical structures’ toxicities.

This study has several limitations. First, the PALN metastases are identified on FDG PET/CT and not by pathologic confirmation. Second, the center of the lymph node does not accurately represent its pathologic growth initial location. Third, inevitable bias exists in the process of delineation of para‐aortic nodal CTV. Last but not least, random and systemic errors might exist because the distance between the nodal center and the blood vessels was manually measured. Although there are several limitations, our study provides the largest data set on the anatomic distribution of FDG‐avid PALNs in cervical cancer and validates NCC of the three mainstream delineation methods on our population and proposes a modified delineation method of para‐aortic nodal CTV.

## 5 CONCLUSION

CTV‐D provided more satisfactory NCC than CTV‐K and CTV‐S. CTV‐M provided the same satisfactory NCC as CTV‐D and reduced the dose to the critical structures.

## CONFLICT OF INTEREST

The authors declare that they have no conflict of interest.

## ETHICAL APPROVAL

This study was approved by Peking Union Medical College Hospital Ethics Committee (Protocol number S‐K 1694). This study was carried out in accordance with the Declaration of Helsinki. Written informed consent was obtained from all patients prior to enrollment.

## Data Availability

Research data are stored in an institutional repository and will be available upon reasonable request to the corresponding author.
